# High-Property Refractive Index and Bio-Sensing Dual-Purpose Sensor Based on SPPs

**DOI:** 10.3390/mi13060846

**Published:** 2022-05-29

**Authors:** Shubin Yan, Pengwei Liu, Zhanbo Chen, Jilai Liu, Lifang Shen, Xiaoyu Zhang, Jiaming Cui, Tingsong Li, Yang Cui, Yifeng Ren

**Affiliations:** 1 School of Electrical Engineering, Zhejiang University of Water Resources and Electric Power, Hangzhou 310018, China; lpw18834800530@163.com (P.L.); chen_zb0416@163.com (Z.C.); liujl@zjut.edu.cn (J.L.); shenlf@zjweu.edu.cn (L.S.); cuijm@zjweu.edu.cn (J.C.); cuiy@zjweu.edu.cn (Y.C.); 2 Joint Laboratory of Intelligent Equipment and System for Water Conservancy and Hydropower Safety Monitoring of Zhejiang Province and Belarus, Hangzhou 310018, China; 3 School of Electrical and Control Engineering, North University of China, Taiyuan 030051, China; zhangxiaoyu9725@163.com (X.Z.); lts15296737639@163.com (T.L.); renyifeng126@126.com (Y.R.)

**Keywords:** surface plasmon resonance, metal-insulator-metal, multimode interference coupling mode theory, biosensor

## Abstract

A high-property plasma resonance-sensor structure consisting of two metal-insulator-metal (MIM) waveguides coupled with a transverse ladder-shaped nano-cavity (TLSNC) is designed based on surface plasmon polaritons. Its transmission characteristics are analyzed using multimode interference coupling mode theory (MICMT), and are simulated using finite element analysis (FEA). Meanwhile, the influence of different structural arguments on the performance of the structure is investigated. This study shows that the system presents four high-quality formants in the transmission spectrum. The highest sensitivity is 3000 nm/RIU with a high FOM^*^ of 9.7 × 10^5^. In addition, the proposed structure could act as a biosensor to detect the concentrations of sodium ions (*Na^+^*), potassium ions (*K^+^*), and the glucose solution with maximum sensitivities of 0.45, 0.625 and 5.5 nm/mgdL^−1^, respectively. Compared with other structures, the designed system has the advantages of a simple construction, a wide working band range, high reliability and easy nano-scale integration, providing a high-performance cavity choice for refractive index sensing and biosensing devices based on surface plasmons.

## 1. Introduction

Surface plasmon polaritons (SPPs) are a kind of hybrid excited state caused by the local coupling of free electrons and photons on a metal surface, which its field distribution decreases exponentially on both sides of the interface [1,2]. SPPs break through the diffraction limit of traditional optics, and have strong optical field limitation and local field enhancement capabilities, which means that they have important application value in optical integrated circuits [3,4]. Many optical phenomena, such as electromagnetically induced transparency [5] and Fano resonance [6,7,8], have been observed in plasma-waveguide coupling systems. In the propagation process of SPPs, the destructive interference between the continuous odd pattern and the discrete even pattern will produce Fano resonance and sharp asymmetric peaks in the transmission spectrum.

In recent years, various classical waveguide structures based on SPPs—including the metal strip waveguide [9,10], metal slot waveguide [11,12] and hybrid plasmonic waveguide [13,14]—have been designed for the fabrication of various photonic devices. The basic structure of the metal slot waveguide is a metal-insulator-metal (MIM) construction. The design of an MIM waveguide sensor based on SPPs has caught researchers’ extensive attention, owing to its characteristics of high constraint, low-loss, long transmission distance and ease of manufacture. Chen et al. [15] designed a plasmonic waveguide structure consisting of an MIM waveguide side-coupled with two same-stub cavities, with a sensitivity of 1100 nm/RIU and a figure of merit (FOM^*^) of 2 × 10^5^. Wen et al. [16] proposed an end-coupled ring-slot-connected MIM waveguide construction; its sensitivity was 960 nm/RIU and its FOM^*^ was 1.65 × 10^4^. Qiao et al. [17] designed an MIM waveguide configuration consisting of an M-type resonator and a stub, with a sensitivity of 780 nm/RIU and an FOM^*^ of 1.56 × 10^5^. Xiao et al. [18] proposed a tunable plasmonic sensor with resonators in an inverted U-shaped resonator; its sensitivity was 840 nm/RIU and its FOM^*^ was 3.9 × 10^5^. Compared with other structures, the performance of the structure is obviously improved. As shown in Table 1, the performance of the proposed structure is superior to other structures. Additionally, optical functional equipment based on an MIM waveguide structure has been designed, including wavelength-division multiplexers [19], a Bragg reflector [20,21], an optical splitter [22,23], a bio-sensor [24], and so on. Meanwhile, many researchers have designed photodetectors [25] and solar absorbers [26] based on surface plasmon resonance (SPR).

In this paper, a surface plasmon resonance system consisting of two MIM waveguides coupled with a transverse ladder-shaped nano cavity (TLSNC) is presented and investigated. The propagation characteristics of the surface plasmon resonance system are analyzed by using finite element analysis (FEA). The effects of the refractive index of the dielectric and the influences of the geometric parameters of the structure on the transmission characteristics are studied, including the length of the two rectangular cavities on the side, the height of the five perpendicular strip cavities, and the coupling gap between TLSNC and the two MIM waveguides. In addition, the application of the designed structure in bio-sensing is studied in detail.

## 2. Materials and Methods

**Figure 1:**
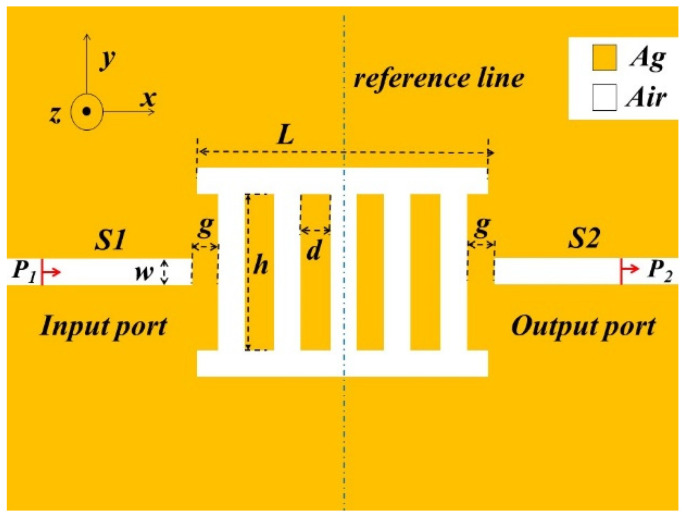
The two-dimensional schematic of two MIM waveguides with a transverse ladder-shaped nano-cavity (TLSNC).

The refractive index sensor structure of a transverse ladder-shaped resonator based on an MIM waveguide is shown in Figure 1. Two MIM waveguides are placed on metallic silver, and a TLSNC is placed between the two waveguides. The yellow area and the white area represent silver and air, respectively. The geometrical analysis model based on FEM was established in order to explore its optical response characteristics. The width *w* of MIM waveguides, the horizontal strip-shaped cavity and the vertical strip-shaped cavity remain constant at 50 nm in order to ensure that only TM_0_ can propagate through the waveguide structure [27]. *d* is the separation distance between two vertical rectangular cavities. The length of the two rectangular cavities on the side is signified as *L*. *h* expresses the height of the five vertical strip-shaped cavities. *g* is defined as the coupling gap between TLSNC and the two MIM waveguides.

The relative permittivity *ε_d_* of air is 1. The permittivity *ε_m_* of Ag is described by the Drude model [28]:

(1) $$\varepsilon_{m}=\varepsilon_{\infty }-\frac{\omega_{p}^{2}}{\omega^{2}+i\omega \gamma }$$

where *ε_∞_* = 3.7 is the boundless frequency permittivity, *ω_p_* = 9.1 eV is the plasma oscillation frequency, *γ* = 0.018 eV is the collision frequency, and *ω* is the circular frequency of the incident light.

The transmission distance of SPPs is generally defined as 1/*e*, which is the propagation length of SPPs. Because the loss of metal is relatively small, the formula is as follows:

(2) $$L\approx \frac{1}{2K_{SPP}^{I}}$$

where *K_SPP_* is the wave vector of the surface plasmon.

The formula of the TM_0_ mode is as follows [29]:

(3) $$\varepsilon_{m}k_{i}\tanh\left(-\frac{jk_{i}\omega }{2}\right)+\varepsilon_{i}k_{m}=0$$

where *k_i,m_* = 2π*/λ* (*ε_i,m_*−1/*n*^2^*_eff_*)^1/2^, and *k_i_* and *k_m_* represent, respectively, the lateral propagation constants of air and silver.

Due to the size of the structure being nanoscale, the contribution of the imaginary part is so small that it can be neglected, such that more energy should be put into the contribution of the real part. Based on the standing wave theory, the resonant wavelengths of the cavity can be expressed by the following formula [30,31]:

(4) $$\lambda =\frac{2Re\left(n_{eff}\right)L_{eff}}{m-\frac{\varphi }{\pi }}$$

(5) $$Re\left(n_{eff}\right)=\sqrt{\varepsilon_{m}-{\left(\frac{k}{k_{0}}\right)}^{2}}$$

where *L_eff_* represents the efficient length of the resonator, *φ* is the phase shift caused by the reflection of SPPs at the dielectric–metal interface, and the positive integer *m* represents the resonance order.

Based on the multimode interference coupling mode theory (MICMT) [32], the transmittance can be deduced as follows [33]:

(6) $$T=\left|\underset{n}{\sum }\frac{2\gamma_{n1}e^{i\varphi_{n}}}{-i\left(\lambda -\lambda_{n0}\right)\tau_{n}+2+\frac{\tau_{n}}{\tau_{n0}}}\right|,\;\varphi_{n}=\varphi_{n1}+\phi_{n}$$

where *φ_n_* is the entire coupled resonant phase of the *n*th pattern, which can be approximated as a constant. *ϕ_n_* represents the difference between the output phase and the input phase of the *n*th resonance mode, and *φ_n_* is the coupled phase of waveguide S1 and the *n*th mode in the resonant cavity. *τ_n_*_0_ expresses the internal loss decay time of the *n*th mode, which here is *τ_n_ = τ_n_*_1_ *= τ_n_*_2_, because waveguides S1 and S2 are of equal length and width. *γ_n_*_1_ *≈* 1 is the normalization coefficient, and *λ* and *λ_n_*_0_ represent the incident wavelength and resonance wavelength, respectively

In order to assess the sensing performance of the system, sensitivity (S) and FOM^*^ are introduced, which are defined by the following formula [34,35]:

(7) $$S=\frac{\Delta \lambda }{\Delta n}$$

(8) $$FOM^{*}=\frac{\Delta T}{T\Delta n}$$

where *T* is the transmittance, and Δ*T*/Δ*n* is the transmittance variation caused by the change of a refractive index.

## 3. Simulations and Results

**Figure 2:**
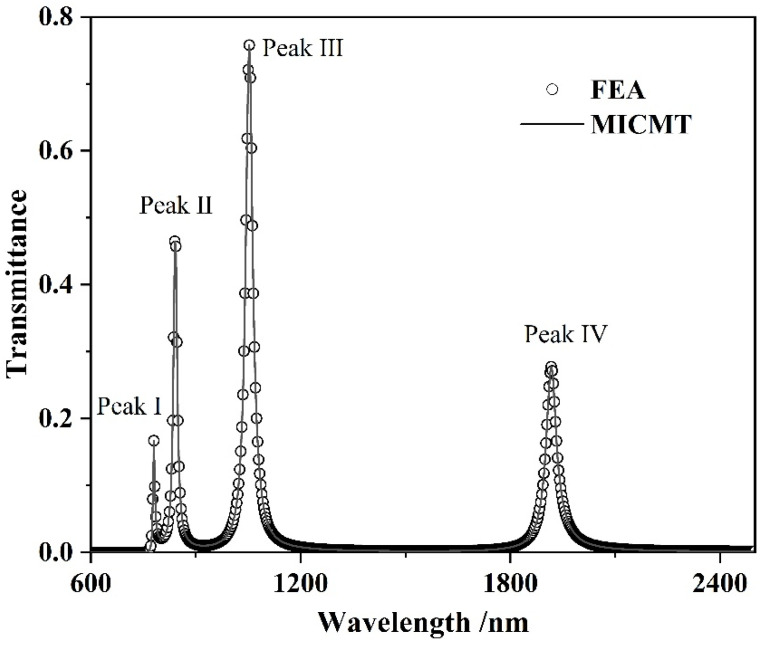
Comparison between the simulation results and theoretical calculation results of MICMT.

COMSOL Multiphysics software (COMSOL Inc., Stockholm, Sweden) was used to establish the geometric analysis model for the optical response characteristics of the decoupled structure. The magnetic field characteristics of the two-dimensional model do not differ seriously from those of a three-dimensional structure [36]. Therefore, in order to save memory and reduce complexity, this paper adopts the two-dimensional model for simulation analysis. The FEA was used to analyze the propagation properties. The geometric parameter settings are as follows: *L* = 510 nm, *h* = 200 nm, *d* = 50 nm, *w* = 50 nm and *g* = 10 nm. The normalized transmittance of the waveguide configuration is described as the quotient of the energy flow between the output end and the input end. The comparison between the simulation results and theoretical calculation results of MICMT is shown in Figure 2. As shown in Figure 2, the graph confirmed that the simulation results of FEA and the theoretical calculation results of MICMT were a match. At the same time, it can be seen that four high-quality resonance peaks—named Peak I, Peak II, Peak III and Peak IV—appear in the transmission spectrum of the proposed structure. The four resonance peaks enhance the ability of the system to eliminate interference factors.

**Figure 3:**
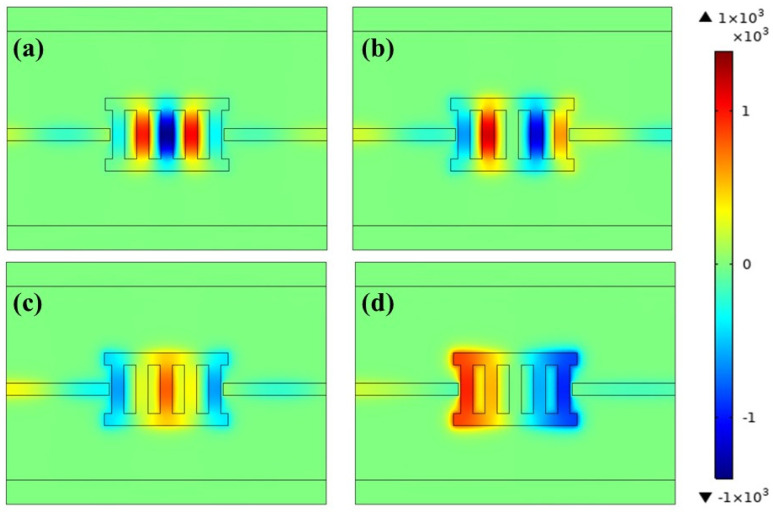
The magnetic field *| H_z_ |* of: (**a**) Peak I (*λ* = 780 nm), (**b**) Peak II (*λ* = 843 nm), (**c**) Peak III (*λ* = 1053 nm) and (**d**) Peak IV (*λ* = 1917 nm).

In order to understand the physical reason for the four resonance peaks more comprehensively and deeply, the magnetic field distributions of four transmission peaks at their wavelengths (*λ* = 780 nm, 843 nm, 1053 nm and 1917 nm) were drawn. All of the magnetic field diagrams are normalized for research convenience. As shown in Figure 3, standing wave resonance occurs in the TLSNC and MIM waveguides of the four modes, such that the incident light can pass through the resonant cavity and exit to form resonant peaks. It was found that, compared with Peak IV, Peak III has weaker magnetic field distribution in the TLSNC and stronger magnetic field distribution on the waveguide, as displayed in Figure 3c,d. As shown in Figure 2, the transmission peak amplitude of Peak IV is less than that of Peak III. This shows that when the energy of the TLSNC is divided more, the energy of the exit waveguide is divided less, resulting in a smaller transmission peak.

**Figure 4:**
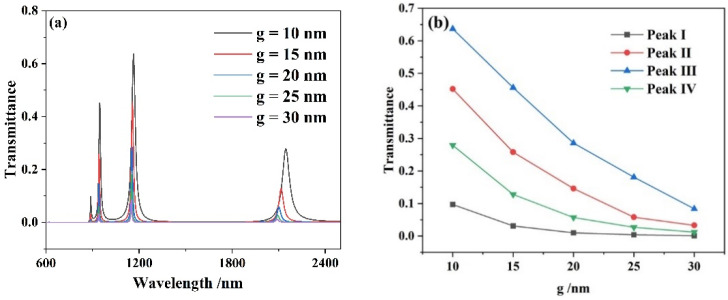
(**a**) Transmission spectra of the complete structure for diverse coupling gap *g*; (**b**) varying transmittance with the increasing coupling gap.

Considering the practicability of the whole system, firstly, the influence of coupling gap *g* on the system performance is studied by changing the *g* from 10 to 30 nm at an interval of 5 nm. As represented in Figure 4, with *g* increased, the blue shift appears in the transmission spectra in a very small range, which is almost negligible, but the transmittance of the transmission peak decreases sharply. This can be explained by the fact that when the coupling gap *g* between the TLSNC and MIM waveguides S1 and S2 increases, the effective coupling distance of the system decreases, the transmission efficiency of the overall structure decreases, and the transmittance of the system decreases. The coupling gap *g* determines the bottleneck of the whole system, which can be seen from the influence of the coupling distance on the transmission spectra of the whole system.

**Figure 5:**
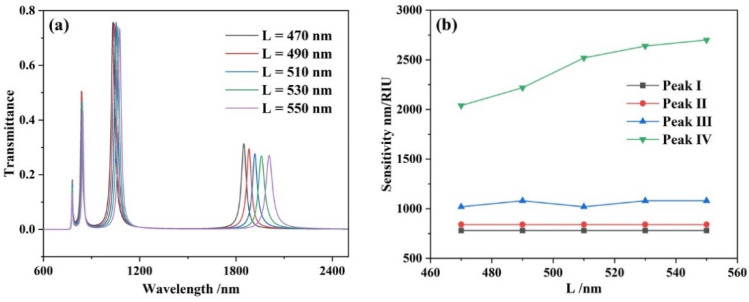
(**a**) Transmission spectra of the complete system for various lengths of *L* of the two rectangular cavities on the side; (**b**) varying sensitivities with the increasing lengths of the two rectangular cavities on the side.

In order to investigate the influences of the diverse lengths of the two rectangular cavities on the side, *L* was increased from 470 nm to 550 nm in steps of 20 nm. Other arguments were set as *h* = 200 nm, *d* = 50 nm, *w* = 50 nm and *g* = 10 nm. As shown in Figure 5a, with the increase of *L*, the transmission spectra of the four resonance peaks show different degrees of red shift. This phenomenon could be explained by analyzing the magnetic field distribution. As shown in Figure 3a, the magnetic field energy of Peak I has a distribution mainly in the perpendicular rectangular cavity, while there is only a very weak magnetic field distribution in the horizontal cavities on both sides. In Figure 3d, Peak IV has a strong magnetic field energy distribution in two horizontal cavities. This leads to the large redshift of Peak IV, while the redshift of Peak I is almost negligible.

**Figure 6:**
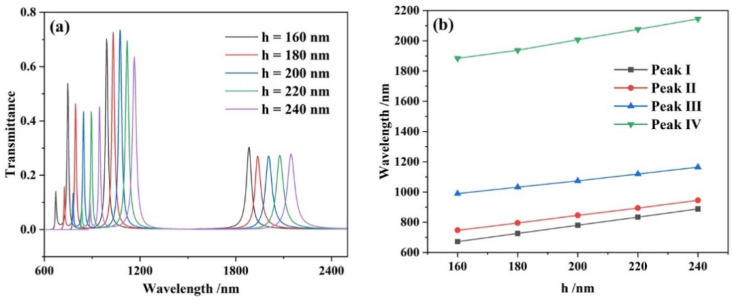
(**a**) Transmission spectra for different heights *h* of the five vertical rectangular cavities; (**b**) varying wavelengths with the increasing lengths of the five vertical rectangular cavities.

Afterwards, the effects of the height *h* of the five vertical rectangular cavities on the performance of the sensor system were analyzed at 160, 180, 200, 220 and 240 nm, while setting *L* as 550 nm and keeping the other parameters the same. The simulation result shows that with the increase of *h*, there were four obvious linear red-shifts on the transmission spectra of the four resonance peaks, which are represented in Figure 6.

**Figure 7:**
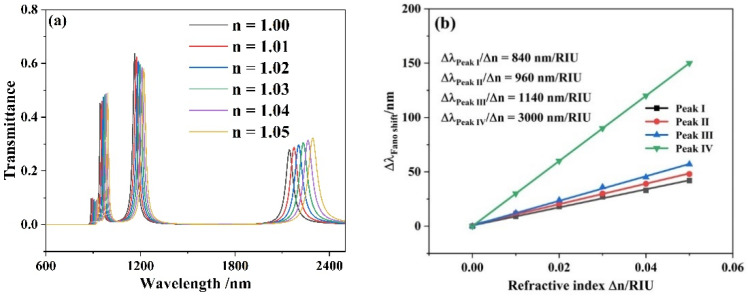
(**a**) Transmission spectra for different refractive index *n* values; (**b**) the change of the peak wavelength with the different refractive indexes.

The resonance system is greatly affected by the change of refractive index *n* of the insulator. Hence, in order to further investigate how the various refractive indexes affect the system performance, *n* was set to increase from 1.00 to 1.05 RIU at an interval of 0.01 RIU. The transmission spectra were displayed in Figure 6a. The parameters of the structure were as follows: *L* = 550 nm, *h* = 240 nm, *d* = 50 nm, *w* = 50 nm and *g* = 10 nm. As shown in Figure 7a, with increases in *n*, the transmission spectra of four resonance peaks have approximately equidistant red shifts. In Figure 7b, the sensitivities of four resonance peaks were calculated to be 840, 960, 1140 and 3000 nm/RIU, respectively. The highest FOM^*^ obtained at *λ* = 888 nm was 9.7 × 10^5^.

## 4. Application in Bio-Sensing

**Figure 8:**
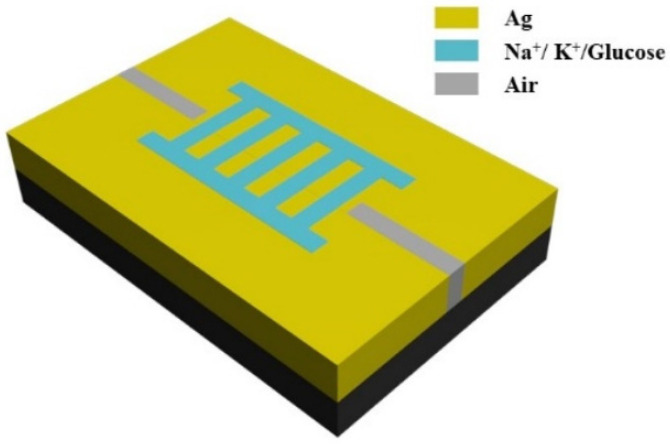
Three-dimensional schematic of the biosensor.

The proposed structure can also serve as a biosensor. There are many patents for biosensors based on surface plasmon resonance [37,38,39]. In human body fluid, sodium ions (*Na*^+^) are the most important electrolyte in extracellular fluid, and potassium ions (*K^+^*) are the most important electrolyte in intracellular fluid. *Na*^+^ and *K*^+^ are of great significance in order to maintain the osmotic pressure, body fluid volume and acid-base balance of normal body fluid [40]. For patients with diabetes, monitoring blood glucose levels is also particularly important. In order to evaluate the detection performance, the TLSNC with the proposed structure was filled with diverse concentrations of *Na*^+^*, K*^+^ and glucose solution. The schematic diagram of the three-dimensional model is shown as Figure 8. The yellow part, blue part, grey part and black part represent silver, filling solution, air and the substrate, respectively. Silver is used as the designated metal in order to capitalize on its enhanced filed penetration, lower ohmic loss, and lesser bandwidth than other noble metals.

The relationships of the refractive index with the concentration variation of *Na*^+^ (mgdL^−1^), *K*^+^ (mgdL^−1^) and the glucose solution (mgdL^−1^) at a constant temperature are as follows [41,42]:

(9) $$n_{Na^{+}}=1.3373+1.768\times 10^{-3}\frac{C\times k}{393}-5.8\times 10^{-6}{\left(\frac{C\times k}{393}\right)}^{2}$$

(10) $$n_{K^{+}}=1.3352+1.6167\times 10^{-3}\frac{C\times k}{529.8}-4\times 10^{-7}{\left(\frac{C\times k}{529.8}\right)}^{2}$$

(11) $$n_{glucose}=1.33230545+0.00011889\times C\times k$$

where *C* expresses the concentration in mgdL^−1^, and *k* is the concentration element. The concentration element *k* for *Na*^+^, *K*^+^, and the glucose solution is 30, 50, and 10, respectively.

The sensitivity equation can be expressed as follows:

(12) $$S_{C}=\frac{\Delta \lambda_{C}}{\Delta C}$$

**Figure 9:**
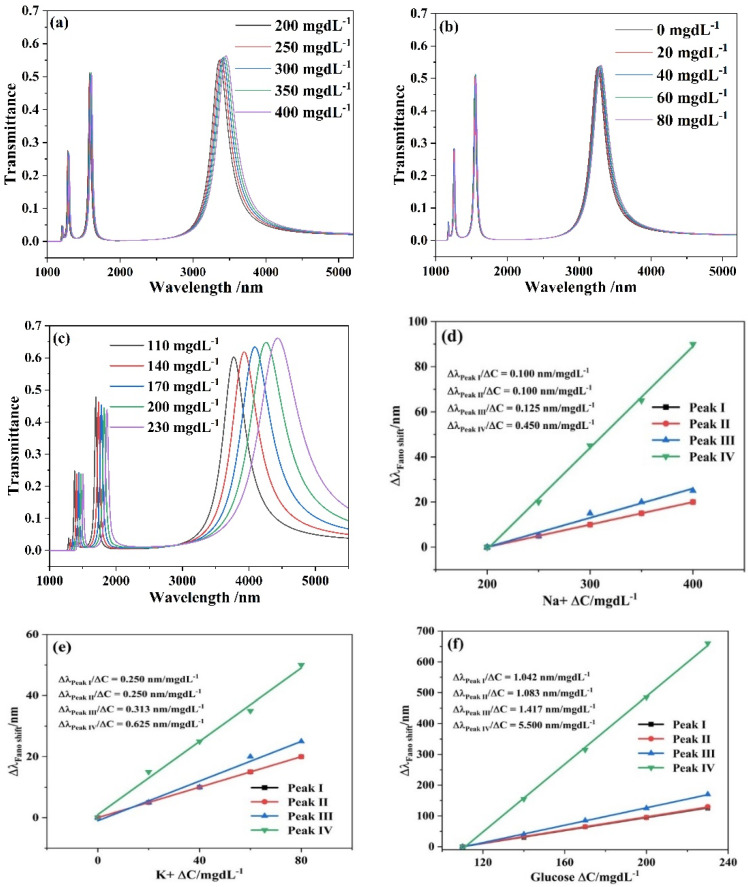
Transmission spectra for different concentration *C* of: (**a**) *Na*^+^; (**b**) *K*^+^; (**c**) glucose solution; the change of the peaks’ wavelength with the diverse refractive index of: (**d**) *Na*^+^; (**e**) *K*^+^; (**f**) glucose solution.

The structural parameters were fixed at *L* = 550 nm, *h* = 240 nm, *d* = 50 nm, *w* = 50 nm and *g* = 10 nm. The concentration of *Na*^+^ was set to 200, 250, 300, 350 and 400 mgdL^−1^; the concentration of *K*^+^ was set to 0, 20, 40, 60 and 80 mgdL^−1^. The glucose solution’s concentration was changed from 110 mgdL^−1^ to 230 mgdL^−1^, with an interval of 30 mgdL^−1^. Their transmittance curves are presented in Figure 9. As shown in Figure 9a–c, distinguishable transmission peaks were observed as the concentrations of *Na*^+^*, K*^+^ and the glucose solution varied with the patient’s range of possibilities. As the concentration of *Na*^+^*, K*^+^ and the glucose solution changed by 1 mgdL^−1^, the observed maximum transmission peak displacements were 0.45 nm, 0.625 nm and 5.5 nm, respectively, which are expressed in Figure 9d–f. Such large transmission peak changes can be easily detected by modern spectrometers. The biosensor model has the advantages of simple construction, fast response, high reliability and easy nano-scale integration, providing a high-performance cavity choice for biosensing devices based on surface plasmons. At the same time, it has certain guiding significance to the development of the real-time monitoring field.

## 5. Conclusions

Herein, a high-performance plasma resonance sensor structure consisting of two MIM waveguides coupled with a transverse ladder-shaped nano-cavity (TLSNC) was designed. Its transmission characteristics were analyzed and simulated, respectively, using multimode interference coupling mode theory (MICMT) and finite element analysis (FEA). The influence of different structural parameters on the performance of the structure was investigated, including the length of the two rectangular cavities on the side, the height of the five perpendicular rectangular cavities, and the coupling gap between the TLSNC and the two MIM waveguides. The simulation results reveal that the structure has four high-quality formants in the 600–2500 nm operating range. The optimal performance was achieved when the sensor structure parameters were set as follows: *L* = 550 nm, *h* = 240 nm, *d* = 50 nm, *w* = 50 nm and *g* = 10 nm. The highest sensitivity was 3000 nm/RIU with a high FOM^*^ of 9.7 × 10^5^. In addition, the proposed structure could act as a biosensor to detect the concentrations of sodium ions (*Na*^+^), potassium ions (*K*^+^) and the glucose solution with maximum sensitivities of 0.45, 0.625 and 5.5 nm/mgdL^−1^, respectively. Compared with other structures, the designed structure has the advantages of simple structure, a wide working band range, strong anti-interference ability and easy nano-scale integration, which means that it has important guiding significance in the optical integrated circuit, refractive index sensing and nano-biosensing fields.

## Figures and Tables

**Table 1:** Performance comparison of various plasmonic sensors.

Reference	Sensitivity (nm/RIU)	FOM*
This paper	3000	9.7 × 10^5^
Chen et al. [15]	1100	2 × 10^5^
Wen et al. [16]	960	1.65 × 10^4^
Qiao et al. [17]	780	1.56 × 10^5^
Xiao et al. [18]	840	3.9 × 10^5^

## Data Availability

Not applicable.
